# Leiomyomatosis-like lymphangioleiomyomatosis: A case report of the colonic manifestation of tuberous sclerosis

**DOI:** 10.1097/MD.0000000000027723

**Published:** 2021-12-17

**Authors:** Ergin Erginoz, Halit Eren Taskin, Gokce Hande Cavus, Abdullah Kagan Zengin

**Affiliations:** a Department of General Surgery, Istanbul University Cerrahpasa – Cerrahpasa School of Medicine, Istanbul, Turkey; b Department of Pathology, Istanbul University Cerrahpasa – Cerrahpasa School of Medicine, Istanbul, Turkey.

**Keywords:** lymphangioleiomyomatosis, neurocutaneous syndrome, perivascular epithelioid cell tumors, tuberous sclerosis

## Abstract

**Introduction:**

Tuberous sclerosis complex is an inherited multisystemic disorder with manifestations in various organ systems as a result of a mutation of 1 of 2 tumor suppressor genes, tuberous sclerosis complex-1 or tuberous sclerosis complex-2. Perivascular epithelioid cell tumors have been shown to be associated with these gene mutations and include a variety of tumors such as angiomyolipomas and lymphangioleiomyomatosis.

**Patient concerns:**

In this report, we present a case of a 28-year-old woman presenting with symptoms of severe abdominal pain and nausea with a medical history of cardiac rhabdomyoma, adenoma sebaceum, Ash leaf spots, bilateral renal angiomyolipomas, and retinal hamartoma, which are manifestations of tuberous sclerosis complex. The patient was operated twice for colonic perforations in the rectosigmoid and ileocecal regions where the pathologic examination revealed multiple tumoral lesions in both specimens.

**Diagnosis:**

The tumor consisted of a myomatous component where the nodules were composed of spindle cells with fascicular array, and a lymphangiomatous component where epithelioid cells could be observed. Immunohistochemically, smooth muscle markers (desmin and SMA) were positive and the epithelioid component showed HMB-45 positivity. A diagnosis of leiomyomatosis-like lymphangioleiomyomatosis was established due to its morphological and immunohistochemical features, the presence of the tumor in multiple foci, and widespread lymphovascular invasion.

**Interventions:**

The patient had a perforation in her bowel twice during the hospital stay and underwent Hartmann operation and ileocecal resection in 2 different surgical operations.

**Outcomes:**

After the second operation the patient developed fever and was diagnosed with SARS-CoV-2 infection. No other complication was observed during her stay and the patient's follow-up was unremarkable.

**Conclusion:**

Perivascular epithelioid cell tumors are associated with tuberous sclerosis and can rarely appear in the colon. Therefore, lymphangioleiomyomatosis should be in the differential diagnosis in a tuberous sclerosis patient presenting with a colonic tumor.

## 1 Introduction

Tuberous sclerosis complex (TSC) is an autosomal dominant inherited neurocutaneous syndrome with manifestations in various organ systems due to a loss-of-function mutation in the TSC-1 or TSC-2 genes.^[1]^ The brain, heart, kidneys, lungs, and skin are the most commonly involved organs and the gastrointestinal manifestation of TSC usually includes hamartoma formation and polyposis.^[2]^ The TSC often gives rise to perivascular epithelioid cell tumors such as renal angiomyolipomas and lymphangioleiomyomatosis (LAM) of the lung.^[3]^ Gastrointestinal involvement, especially LAM formation, is quite rare in TSC and only 2 cases have been previously reported in the literature.^[3,4]^ In this report, we present a case with leiomyomatosis-like LAM in both the ileocecal and rectosigmoid locations of the gastrointestinal system.

## 2 Methods

The ileocecal and rectosigmoid resection specimens were fixated in formalin. The specimens were prepared for histopathological examination with the standard paraffin technique and routine hematoxylin & eosin staining. The 3,3′-Diaminobenzidine detection kit was used for immunohistochemical staining. The primary antibodies including clone, dilution, and manufacturer were Desmin (DE-R-11, RTU, Ventana), SMA (1A4, RTU, Cell Marque), CD34 (QBEnd/10, RTU, Ventana), DOG-1 (Sp31, RTU, Cell Marque), c-KIT (EP10, RTU, Ventana), S100 (S100, RTU, Ventana), ER (SP1, RTU, Ventana), PR (1E2, RTU, Ventana), and HMB-45 (gp100, RTU, Ventana). Convenient positive and negative controls were used. Ethics approval was not required for this report and written informed consent form was obtained from the patient.

## 3 Case report

### 3.1 Clinical findings

**Figure 1:**
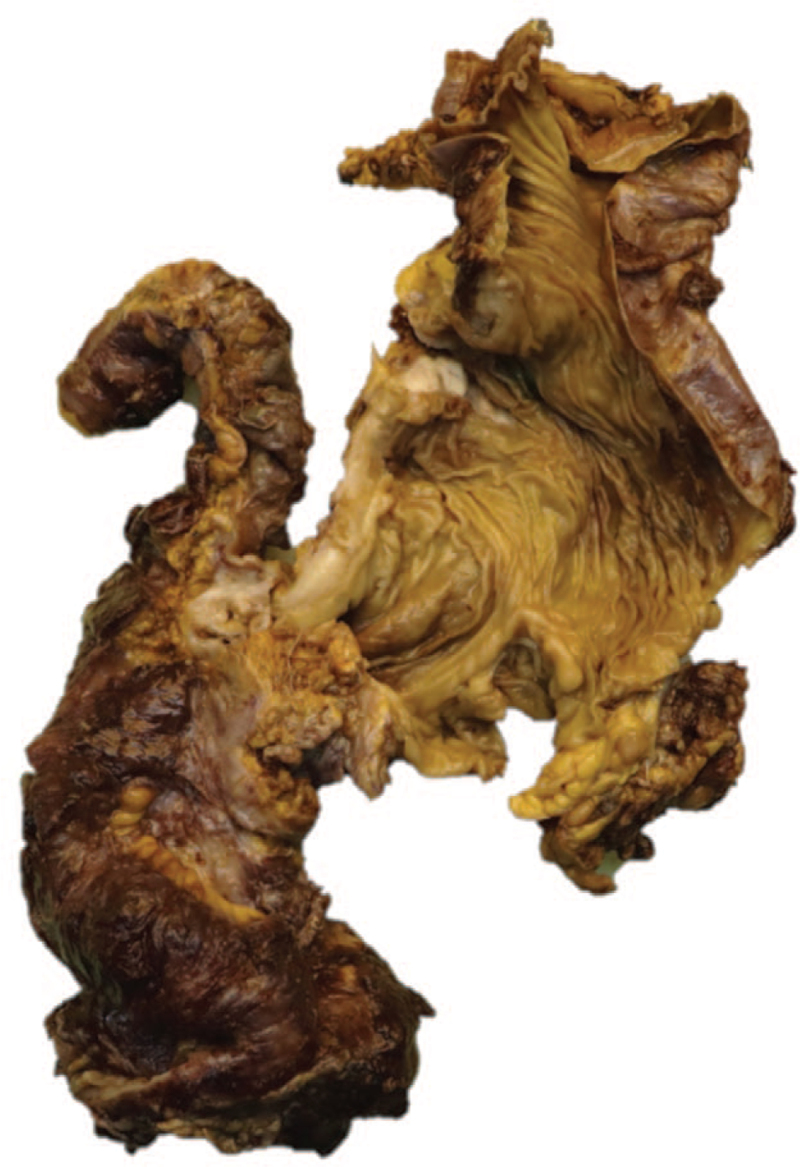
Diffuse nodular wall thickening of the colon in ileocecal resection specimen.

A 28-year-old woman presented to the emergency department with severe lower abdominal pain that was associated with nausea. Two weeks prior to admission, she had symptoms of abdominal pain and bloody diarrhea which was later diagnosed to be amebiasis. The patient was diagnosed with tuberous sclerosis at the age of 6 months when she developed arrhythmia related to a cardiac rhabdomyoma. The rest of the medical history included adenoma sebaceum, Ash leaf spots, bilateral renal angiomyolipomas, left-sided retinal hamartoma, and allergic rhinitis. She was previously operated 3 times for the removal of multiple renal angiomyolipomas and underwent partial cystectomy due to invasion of the tumor. Physical examination was normal. The laboratory work-up was unremarkable except for anemia (hemoglobin 9.6 g/dL) and an elevated C-reactive protein value of 125 mg/dL. The computerized tomography (CT) scan revealed extralumination of the rectal contrast material at the rectosigmoid region, indicating bowel perforation. The patient underwent proctosigmoidectomy and formation of an end colostomy together with the closure of the anorectal stump (Hartmann operation). Two weeks after the operation, the patient developed a new onset abdominal pain. The CT scan revealed contrast extralumination in the distal ileum and the patient underwent ileocecal resection due to a new onset bowel perforation and severe adhesions. Several days after the second operation she developed fever and was diagnosed with SARS-CoV-2 infection for which she took treatment. Both of the rectosigmoid resection specimen and the ileocecal resection specimen were reported as leiomyomatosis-like lymphangioleiomyomatosis (Fig. 1). After 8 months of follow-up, the patient's rectoscopic examination together with a control CT scan was normal. At the first year of follow-up, the patient had an ileostomy closure surgery.

### 3.2 Histopathological findings

Grossly, the rectosigmoid specimen (16 cm in length, 7 cm in width, 1 cm in thickness) consisted of full-thickness hemorrhage extending 7 cm proximally from the surgical margin. Macroscopically, there was a 3 cm perforation area in the central part of this hemorrhagic region. Pseudopolypoid lesions ranging from 0.1 to 0.3 cm in diameter were observed in the surrounding mucosa. The ileocecal resection material (ileum 4 cm and colon 16 cm in length) consisted of a 5 cm, pale-colored, nodular lesion in the cecum. Numerous pale nodular lesions, ranging from 0.3 to 5 cm in diameter extending from the mucosa to the subserosa were observed in the cecum. In all resected specimens, the intestinal wall was diffusely thickened and nodular. Infiltration into the subserosal adipose tissue was observed.

**Figure 2:**
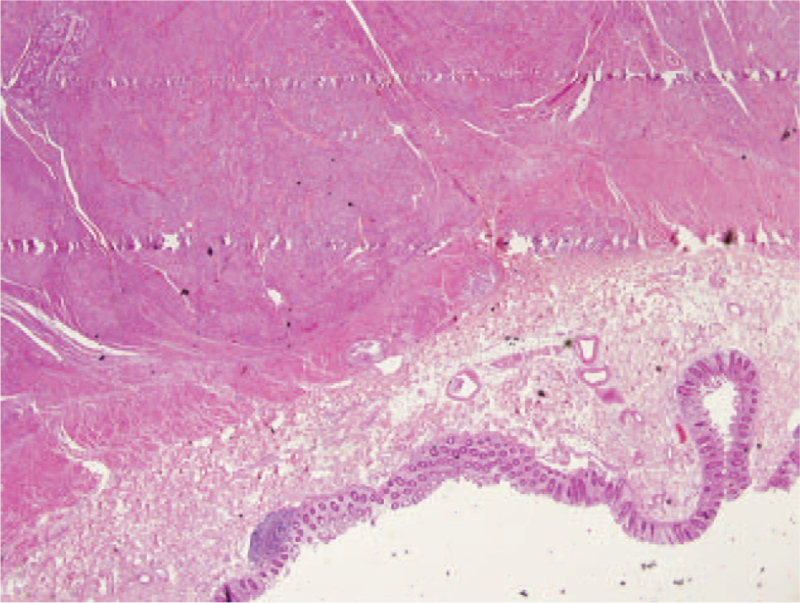
Spindle cell nodular lesion extending into the lamina propria in the colonic wall. (H&E 20× magnification).

**Figure 3:**
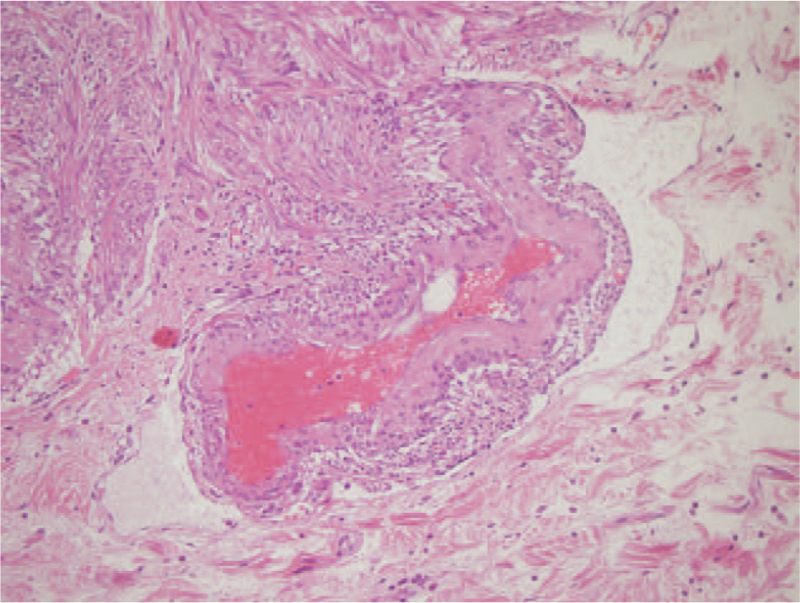
Epithelioid cells clustered around dilated vascular structure. (H&E 400× magnification).

**Figure 4:**
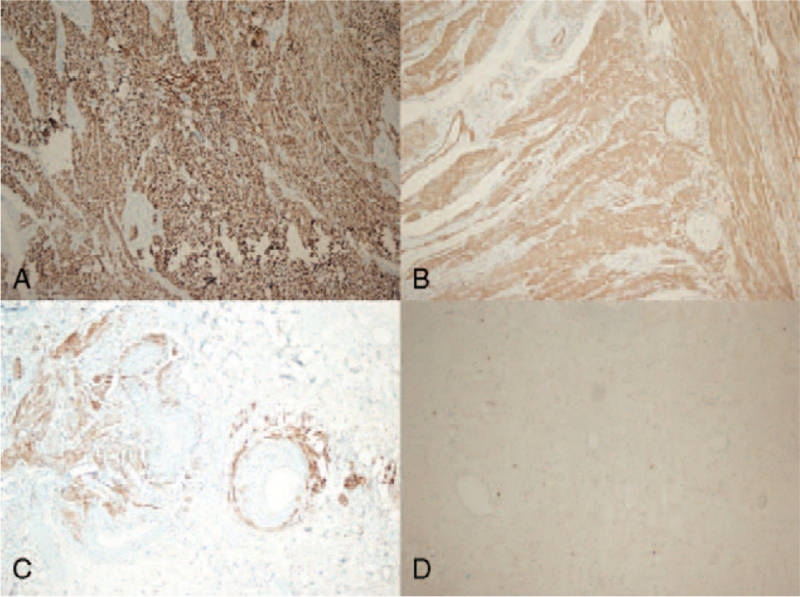
(A) Desmin and (B) SMA were positive in spindle and epithelioid cells immunohistochemically (100× magnification). (C) HMB-45 was positive only in epithelioid cells lined up around vascular structures (200× magnification). (D) C-KIT was negative in all nodular lesions.

Histological examination revealed nodular lesions with an extensive growth pattern, extending from the submucosa to the mesorectal adipose tissue in some areas, without affecting the mucosa. Microscopically, the nodules consisted of spindle cell bundles with prominent elongated nuclei organized into branching short fascicles (Fig. 2). Necrosis, pleomorphism, and mitotic activity were not observed. Among the spindle cells, clusters of epithelioid cells were observed around dilated vascular structures lined with endothelium in some areas (Fig. 3). It was noted that these epithelioid cell assemblages in the vessel walls tended to merge with the surrounding lymphatics. Mature adipose tissue was not observed. In the surrounding intestine where the tumor was absent, severe active colitis findings were observed. Immunohistochemically, the spindle and epithelioid cells in both of the resected specimens stained positive for desmin, SMA, and HMB45 and negative for CD34, DOG-1, c-KIT, S100, ER, and PR (Fig. 4). The Ki-67 proliferation index was found to be 3%. There was no evidence of lymphatic or vascular invasion.

## 4 Discussion

Leiomyomas are benign stromal tumors characterized by smooth muscle proliferation that are frequently observed in the extremities and arise from the deep subcutis or skeletal muscle.^[5]^ In the gastrointestinal tract, they are most commonly seen in the esophagus and anorectum.^[4]^ They are often differentiated from other spindle cell mesenchymal neoplasms such as gastrointestinal stromal tumor (GIST) by immunophenotyping. GISTs are positive for CD34, CD117, and DOG-1, while leiomyomas are negative with immunohistochemical staining.^[6]^

Leiomyomatosis refers to the proliferation of smooth muscle cells forming focal tumors at various organ systems throughout the body. Perivascular epithelioid cell tumors (PEComa) are an expanding family of tumors that differ from each other morphologically and immunohistochemically. This tumor family includes angiomyolipoma, lymphangioleiomyomatosis, clear cell sugar tumor of the lung, and a number of spindled and epithelioid neoplasms.^[7]^ Lymphangioleiomyomatosis is a rare tumor that is caused by the proliferation of perivascular epithelioid cells around lymph nodes and interstitial lymphatics.^[8]^ While lymphangioleiomyomas express localized lesions, lymphatic chain involvement in large segments is referred to as lymphangioleiomyomatosis.^[7]^

PEComas are usually associated with TSC-2 and rarely TSC-1 loss-of-function mutations. These mutations lead to the activation of the mammalian target of rapamycin pathway.^[9–11]^ In addition, p53 mutations can be seen frequently in TSC-2 mutant malignant PEComas.^[9,12]^ Infrequently, TSC-2 non-mutant PEComas shelter translocations containing transcription factor binding to IGHM Enhancer 3.^[9]^

In a patient with tuberous sclerosis, gastrointestinal leiomyomatosis-like LAM is extremely rare and to our knowledge only 2 reports involving the large bowel have been previously reported in the literature.^[3,4]^ Goh et al^[4]^ reported leiomyomatosis-like LAM of the ascending, transverse, and descending colon along with bilateral renal angiomyolipoma in a female patient with tuberous sclerosis complex. Similar to our findings, the smooth muscle cells stained positive for HMB45 and negative for CD34 and CD 117, which suggested leiomyomatosis-like LAM. Kolin et al^[3]^ described a case with tuberous sclerosis complex with a synchronous L-cell type rectal neuroendocrine tumor and leiomyomatosis-like LAM of the rectum. In the report, the immunostaining of the leiomyomatosis-like LAM lesion showed positivity for desmin, caldesmon, HMB-45, and CD117, and showed negativity for CD34.^[3]^

The tumors in our case consisted of both spindle cell proliferation with fascicular array and elongated nuclei and perivascular epithelioid groups. The myomatous component also infiltrated the lymphovascular structures in some areas. Mitotic activity and nuclear pleomorphism were not observed in the cells forming the tumor. In the spindle cell component, smooth muscle markers were positive and GIST markers were negative. HMB-45 expression was observed in the epithelioid component. As a result of these findings, we interpreted our case as leiomyomatosis-like lymphangioleiomyomatosis since the lesions were observed simultaneously in both rectosigmoid and ileocecal resections and lymphatic invasion was present in large segments.

Since LAM formation in tuberous sclerosis commonly occurs within the lung, treatment is usually directed towards improving lung function. Sirolimus has been shown to slow the decline in lung function in patients with LAM, while everolimus is a second-line agent which is used in the treatment of TSC-related angiomyolipomas and central nervous system tumors.^[13–15]^ Treatment of TSC-related angiomyolipomas should take into account the clinical context and the tumor characteristics and usually include the use of a mammalian target of rapamycin inhibitor together with a nephron-sparing surgery for the conservation of normal renal function.^[16]^ Data regarding the treatment and prognosis of gastrointestinal LAM in TSC patients is lacking but surgery is warranted in the case of an emergency such as bowel perforation observed in this patient.

### 4.1 Consent for publication

Written informed consent was obtained from the patient for publication of the case details and accompanying images.

## Author contributions

**Conceptualization:** Ergin Erginoz, Halit Eren Taskin, Gokce Hande Cavus, Abdullah Kagan Zengin.

**Data curation:** Ergin Erginoz, Halit Eren Taskin, Gokce Hande Cavus, Abdullah Kagan Zengin.

**Formal analysis:** Ergin Erginoz, Halit Eren Taskin, Gokce Hande Cavus.

**Investigation:** Ergin Erginoz.

**Supervision:** Gokce Hande Cavus.

**Visualization:** Ergin Erginoz, Gokce Hande Cavus.

**Writing – original draft:** Ergin Erginoz, Halit Eren Taskin.

**Writing – review & editing:** Ergin Erginoz.
